# The Moderating Role of Gender and Mediating Role of Hope in the Performance of Healthcare Workers During the COVID-19 Pandemic

**DOI:** 10.3390/bs14121167

**Published:** 2024-12-05

**Authors:** Demet Cakiroglu, Selen Yılmaz Isıkhan, Hamit Coskun

**Affiliations:** 1 Vocational and Higher School of Social Sciences, Hacettepe University, 06100 Ankara, Türkiye; seleny@hacettepe.edu.tr; 2 Department of Psychology, Faculty of Arts and Science, Bolu Abant İzzet Baysal University, 14030 Bolu, Türkiye; hamitcoskun2000@gmail.com

**Keywords:** resilience, hope, individual performance, psychological capital, role of gender, health workers, nurses, COVID-19, pandemic

## Abstract

This study aimed to examine the relationship between resilience and the hope levels of healthcare workers who have experienced the COVID-19 pandemic, the effects of resilience and gender on individual performance, the effects of hope levels on individual performance, and aimed to determine whether hope mediated the effect of resilience on individual performance. This was a descriptive cross-sectional study. The psychological resilience, hope, and individual performance of healthcare workers affiliated with the Istanbul Provincial Health Directorate were obtained from online questionnaires completed by participants between April 2021 and August 2021. This purpose was served by the Brief Resilience Scale, the continuous hope scale, and the Individual Performance Scales. A hierarchical regression analysis, mediation analysis, and moderation analysis were performed on 412 healthcare workers to test the hypotheses. There was a significant relationship between resilience and hope levels among healthcare professionals. Healthcare professionals’ levels of resilience and hope also had significant effects on their individual performance. The indirect effect of healthcare professionals’ psychological resilience levels on their individual performance through hope was not significant. Gender also had a significant effect on resilience, hope, and agency thinking. Men’s mean scores for resilience, hope, and agency thinking are higher than women’s. The findings show that employees with high hope and resilience are more likely to recover from stressful situations and display better individual performance. This study has significant implications for the advancement of the field of psychosocial assessment of healthcare workers in times of health crisis. It offers both a practical and a theoretical perspective on the effects of hope and resilience on employee performance as psychological capital that can help all healthcare managers and employees, especially in times of crisis such as the COVID-19 pandemic. It can be said that hope is more important than resilience in terms of its propensity to enhance individuals’ performance. Our findings suggest that employees with high levels of hope and resilience are more likely to recover from stressful situations and achieve better personal performance. Organizations should focus more on hope and resilience by promoting positive attitudes among employees and managers to improve their ability to cope with crises.

## 1. Introduction

Healthcare professionals have experienced many more difficulties, including job stress, excessive working hours, role ambiguities, and role conflicts, than anyone else during the COVID-19 pandemic. Among the factors that affect employee performance, psychological resilience and hope are psychological capital. Positive psychological capital is a concept that helps to measure human resources in organizations [1,2]. From this point of view, the research model has been tested on healthcare workers—mainly doctors and nurses—who work as a team in the health sector, where face-to-face interaction is intense and roles are highly interdependent.

Many studies have focused on the relationship between resilience and hope [1,3,4,5,6,7,8,9,10]. Existing studies have generally indicated that there is a positive relationship between hope and performance [2,6,11,12] and between resilience and employee performance [2,7,13,14]. In this context, many studies have focused on psychological capital elements to increase employee performance; however, to our knowledge, few have examined resilience and hope together. The study makes a unique contribution to the field in this respect.

This study has important implications for advancing the field of psychosocial assessment of healthcare workers in times of health crisis. The effects of hope and resilience on employee performance as psychological capital that can help all healthcare managers and employees, especially in times of crisis such as the COVID-19 outbreak, can make many scientific contributions to developing health policy and management strategies. In addition, the fact that the psychological well-being of health workers with psychological resilience and hope will increase the effectiveness not only of their health but also of the general health system can be seen as another social contribution of the study.

The study was conducted between April 2021 and August 2021, when the pandemic was spreading rapidly and the health systems in Türkiye and Istanbul were under great pressure. During these periods, the number of daily cases in Türkiye was between 40,000 and 60,000, while by April 2021, the total number of cases in Türkiye had risen to over 4 million. Looking at the first half of 2021, the number of daily cases in Istanbul was over 10,000, and intensive care bed occupancy rates reached 75–80% [15]. Istanbul, the most populous city in Türkiye, also occupies a very important place in the Turkish economy. The fact that almost 1/5 of the Turkish population lives in Istanbul has led to a high increase in the number of cases. In addition, this increase in the number of cases in Istanbul has put pressure on the health system and has made it the province most affected by the pandemic.

The theoretical basis of this research is based on the hope theory [16] and the job demand resource (JDR) model, which emphasizes that employees need resources to help them cope with their demanding work routines. This research tried to determine whether healthcare workers’ resilience and hope levels would affect their performance.

The aim of this study was to (a) examine the relationship between the resilience and hope levels of healthcare workers who experienced the COVID-19 pandemic, (b) examine the effects of resilience levels on individual performance, (c) examine the effects of hope levels on individual performance, and (d) examine the effects of individual resilience levels on individual performance. This study aimed to investigate whether hope would have a mediating role in its effect on performance and the moderating effect of gender. Resilience through hope may act as buffer in ambiguous situations, such as the COVID-19 pandemic. This study contributes to the field by providing critical information on how managers can strengthen their employees in similar cases.

## 2. Background

The subject of this research was to investigate the effects of psychological resilience and hope levels of health workers who experienced the COVID-19 pandemic on their individual performance. The fact that the study was conducted in Istanbul, the province with the highest number of COVID-19 cases and a higher number of hospitals and healthcare workers than many provinces in Türkiye, has high value in terms of the effect and efficiency of this study. As the first study of this nature in which COVID-19 variants are considered, this study will guide future studies. The research findings will bring a specific perspective to the literature and can be applied to predict, understand, and motivate employee behavior in managing processes in anomalous situations such as pandemics.

### 2.1. Resilience

The concept of resilience is very important for today’s healthcare professionals. Psychological resilience, defined as the capacity for self-recovery in difficult situations and internal and external adaptation and regeneration under these distressing conditions [17], is an effective tool that enables employees to cope with the stressful situations they experience. Employees with high psychological resilience do not regress in the face of distress and failure. As Victor Emil Frankl, a theorist of resilience and Logotherapy [18], has suggested, a person who finds meaning in a situation can overcome any dilemma and achieve success.

Psychological resilience is the capacity to successfully adapt to life, tolerate adversities, and overcome life crises, despite the difficulties caused by adverse life conditions and events [17]. Resilient individuals develop positive emotions over time and use these emotions intelligently. Studies have shown that employees with more resilience respond positively to setbacks and act creatively [19].

### 2.2. Hope

Hope is defined as a cognitive process which helps people to achieve their desired goals, promotes a positive state of motivation [20], and grants one with the will and path planning required to achieve one’s goals [1]. It is much more than a simple desire accompanied by the expectation of fulfillment and consists of two components: alternative ways of thinking and acting thinking [20]. Agency thoughts, namely goal-directed energy or thoughts, motivate and empower the individual in planning and implementation [11], and can include feelings and thoughts about making successful decisions that will allow the person to meet their goals even when they are faced with obstacles [20]. An acting thought, namely specific plans or intentions to meet specific goals, is defined by the desire to achieve a goal and feeling empowered to accomplish that goal [16]. Detailed planning when obstacles arise strengthens alternatives and makes it easier to find new solutions [1,11]. In order for a person to achieve a high level of hope, the perceptions of these two components are expected to be positive [16].

An employee with high hopes has a positive psychological development status in terms of continuing toward his or her goals and successfully working toward said goals when necessary [6]. In addition, they have the energy to think of multiple solutions when faced with problems or difficulties and experience more positive emotions, feeling happy, peaceful, determined, active, energetic, and cheerful in their daily lives [20]. Hope, which includes positive expectations for the future (Snyder et al. [16]), is associated with positive expectations that COVID-19 and similar difficulties will surely pass one day.

### 2.3. Psychological Resilience and Hope

Hope is the driver of resilience because of its importance in enhancing motivation in the face of persistent obstacles [20]. Many studies have stated that hope provides protection against problems encountered during one’s life [16]. Hopeful individuals believe that hopeful thinking will protect them now and in the future. In this way, hope acts as a buffer against unexpected negative effects. In addition, hopeful people can successfully cope with problems in their lives and are aware that they may encounter problems in the future [16].

A study of surgical nurses found a strong relationship between psychological resilience and hope [21]. Many studies have drawn attention to this relationship [2,4,10,11,12]. This had led to the following hypothesis:

**H1:**  *There is a positive relationship between psychological resilience and the hope levels of healthcare workers.*

### 2.4. Gender Difference

Research in psychology has produced a sizable body of findings on gender differences (e.g., [22]). Even though there are many explanations (socio-cultural, evolutionary, brain, and hormonal theories) about gender differences, the selectivity hypothesis has received much attention from researchers. The selectivity hypothesis assumes that compared to males, females tend to process incoming data more comprehensively, and their threshold for apprehending information is lower [23,24,25,26]. In contrast, males are more selective when it comes to processing data and pay attention to the most important stimuli. Males and females also process information differently when experiencing a negative mood. Females tend to use more detailed processing when they are in a sad mood [27]. In contrast, men use distraction strategies to remedy a sad mood.

Psychology studies have focused more on negative emotions such as fear and anxiety [28,29,30,31]. On the other hand, gender differences related to positive behaviors have received little research attention. In recent years, studies have begun to examine the issue of hope and gender differences [32,33]. Longitudinal research has noted a general decline in hope over time, and has observed that it is declining more rapidly in females than males [34]. Even though these studies provided inconsistent findings, gender seemed to interact with hope. Given the importance of cultural differences, this study illuminates this issue in Türkiye, representing both collectivistic and individualistic cultures.

### 2.5. Individual Performance

Employee performance is significant for many organizations because it directly affects organizational performance, which is expressed as business results (efficiency, quality, etc.), market results (sales, market share, customer satisfaction, etc.), and financial results (costs, revenues, profits, etc.) [35].

Employees are influenced by their organization and external environmental factors, especially their individual characteristics, while performing their duties in their organizations. The individual performance of employees emerges as a result of the harmony of these factors. The attitude of each individual in the face of adverse events and troublesome situations in life is different. While some people adapt to stressful living conditions and still have a positive attitude, some individuals have difficulty using this skill and their individual performance suffers as a result. Individual differences may stem from psychological resilience and hope levels.

### 2.6. The Effect of Resilience, Hope and Gender on Individual Performance

In times of crisis and organizational change, sustainable hope is particularly critical for maintaining employees’ well-being, helping them to easily overcome challenges, and facilitating positive organizational change. Uncertainty and the stress it brings can lead to a loss of hope. This can lead to poor productivity and performance throughout the entire organization.

An important aspect of psychological capital in organizational terms is that it can be developed with short-term training and has a positive effect on employees’ job performance [6]. Existing studies have revealed that there is a positive relationship between resilience, hope, and individual performance, and an increase in the level of psychological capital causes an increase in an individual’s performance by creating a motivational effect [2,3,5,6,7,8,9,19]. More recent research showed that high psychological resilience positively affected nurses’ attitudes towards their profession, future, life, work, and life outcomes [14]. Studies also emphasized a positive relationship between resilience and employee performance [2,7,13,14]. This evidence led to the following hypothesis:

**H2:**  *The psychological resilience levels of healthcare professionals have positive effects on individual performance.*

As a psychological force, hope helps individuals cope with stressful situations, makes them more resilient, and improves their well-being and psychological health [16,19]. In this respect, hope has been shown to positively affect individuals’ performance. Individuals with higher levels of hope are more likely to be motivated to work towards their goals and think of ways to achieve a desired goal. Studies have generally found similar results, indicating a positive relationship between hope and performance [1,12], which has led to the following hypothesis:

**H3:**  *Hope levels of healthcare professionals have positive effects on their individual performance.*

When surrounded by problems and difficulties, resilience, known as continuing, returning, and even going beyond to achieve success [2], will lead to an increase in individual performance when combined with the effect of hope, which plays a role in reducing stress in challenging conditions on this path. A person who realizes his or her potential with psychological resilience has the potential to turn a disaster into victory [18]. In this context, it seems plausible that hope has a mediating effect on persevering towards the goals of achieving success and re-planning paths toward goals when necessary. This has informed the following hypothesis:

**H4:**  *The indirect effect of the psychological resilience levels of healthcare professionals on their individual performance through hope is significant.*

More recent studies have begun to examine the issue of hope and gender differences [32,33]. One study has shown that female adolescents score higher for the emotional cutoff, the interpersonal domain, than male adolescents [33]. Given this finding, it seems plausible that

**H5:**  *Females would be expected to achieve a higher performance than males when they have low- or medium-level hope. However, as hope increases, gender differences should disappear. In other words, gender might play a moderating role between hope and performance.*

## 3. The Study

### 3.1. Aims

**Figure 1:**
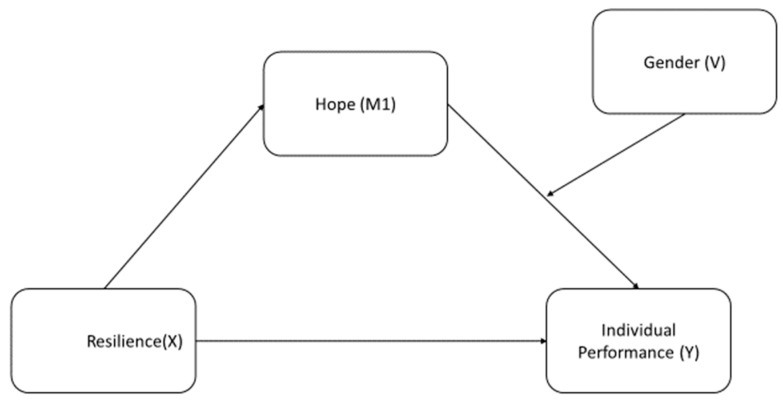
Hypothetical model of the study.

It is critical to understand the basic mechanism that links employees’ positive psychological resources, such as hope and resilience, with their individual performance in anomalous situations such as the COVID-19 pandemic. In this respect, this study aimed to examine the relationship between resilience and the hope levels of healthcare workers who experienced the COVID-19 pandemic, the effects of resilience levels on individual performance, and the effects of hope levels on individual performance, and to investigate the mediating role of hope and the moderating effect of gender with regard to resilience and its effects on individual performance (see Figure 1).

### 3.2. Design

This was a comparative descriptive study.

### 3.3. Sample/Participants

While determining the sample size, the rules of thumb were applied, which proposed that the ratio of the number of individuals (N) to the number of measured variables (p) must be considered [36,37]. In studies involving scales, the recommended N/p ratio as a base ranges from 5 to 10, with a sample size of at least n > 100. A widely accepted ratio is ten cases per indicator variable [37]. On the other hand, when we consider the multivariable regression analysis, one study suggested that the ratio of a sample size to the number of parameters should be 20:1, or at least 10:1 [38]. Therefore, in this study, which included 27 scale questions, six demographic variables, and a total of 33 items (N: p = 412/33= 12.48), a sufficient number for the multivariable regression analysis was obtained. Consequently, 412 healthcare employees who met the inclusion criteria and agreed to participate in the study were included. As an exclusion criterion in the data set of the study, those with less than one year of work experience were not included in the study. Therefore, it was taken into account that each individual had completed the orientation process. Eligible participants were (a) healthcare employees aged 20 years and older; (b) able to speak and understand the Turkish language. The target audience was Istanbul Provincial Health Directorate healthcare workers and persons working in an administrative position in a health center.

### 3.4. Data Collection

Data were collected between April and August of 2021. Researchers applied to the Hacettepe University Ethics Committee after obtaining the necessary approval and permission from the Ministry of Health. Permission was also obtained from the Istanbul Provincial Health Directorate. Questionnaires relating to intensive working and hygiene conditions during the pandemic were collected electronically. Sufficiently clear and understandable explanations were given to the participants regarding the nature of this research. The questionnaire was shared with all employees through the Istanbul Provincial Health Directorate unit officials. The research data were voluntarily applied to the participants.

#### 3.4.1. Demographic Characteristics of Healthcare Professionals and Evaluation Form

The questionnaire used in the research consisted of four parts and included 33 questions. The first part of the research questionnaire consisted of six questions relating to the demographic information of the participating employees. The short resilience scale in the second section consisted of six questions, the continuous hope scale in the third section consisted of 12 questions, and the individual performance scale in the last section consisted of nine questions.

#### 3.4.2. Brief Resilience Scale

The Brief Resilience Scale includes the “Short Psychological Resilience Scale”, which was developed by Smith et al. [39] and translated into Turkish by Doğan [40]. The scale is on a five-point Likert scale, and questions 2, 4, and 6, which had negative meanings in the resilience scale consisting of six statements and a single sub-dimension, were reversed in coding. Cronbach’s alpha coefficient was 0.87.

#### 3.4.3. Continuous Hope Scale

The continuous hope scale consists of 12 items and two subdimensions developed by Snyder et al. [20]. Each of the sub-dimensions, alternative ways of thinking and acting thinking, is measured with four items. Of these four items, one includes statements related to the past, two include statements related to the present, and one includes statements related to the future. The other four items consist of filler statements that are unrelated to hope. While scoring the scale, no points were given to the filling items, and the scores obtained from the alternative ways of thinking and acting thinking sub-dimensions were summed to obtain the continuous hope scale total score. The Turkish adaptation of the scale was carried out by Tarhan [41]. For the hope scale, which consists of two sub-dimensions and has questions scored on an eight-point Likert scale, reverse coding was also performed for questions 3, 5, 7, and 11, which had negative meanings. In this study, Cronbach’s alpha value for hope was 0.86.

#### 3.4.4. Individual Performance Scale

In the one-dimensional nine-variable scale created in the study by Schepers [42], there are nine dimensions of responsibility related to work performance, in which the individual evaluates himself. This scale aims to measure the individual’s own performance based on different aspects, and was previously used to measure work performance in a validity and reliability study conducted by the authors of [43]. In this study, Cronbach’s alpha was 0.88.

### 3.5. Ethical Considerations

This research was approved by the Hacettepe University Senate Ethics Committee (Decision Number: E-21417490-299-00001530098, dated 13 April 2021) and by the T.R. Ministry of Health General Directorate of Health Services. This study was conducted in accordance with the Declaration of Helsinki’s code of ethics. In addition, all healthcare workers participating in this study were informed that all their ethical rights would be protected, and informed consent was obtained prior to their participation in the study. An online survey method was used as a data collection tool.

### 3.6. Data Analysis

Data analyses were performed using the statistical package program IBM SPSS version 24.0. Mediation analyses were performed by installing PROCESS v3.5 by Andrew F. Hayes with SPSS Statistics 24, a mediator analysis add-on. The normality of the data was evaluated using the Kolmogorov–Smirnov test and histogram plots. The assumptions of the multiple regression analysis were also tested. The data met the assumptions of normality, linearity, homoscedasticity, and multicollinearity. Descriptive statistics were presented as the frequency and percentage for categorical variables, the mean and standard deviation for normal distributed numerical variables, and the median (interquartile range) for non-normally distributed numerical variables. Pearson correlation coefficients between resilience, hope, and individual performance variables were computed. For interpretation, Pearson correlation values <0.10 were considered to indicate negligible correlation, values between 0.10 and 0.39 indicated weak correlation, values between 0.40 and 0.69 indicated moderate correlation, values between 0.70 and 0.89 indicated strong correlation, and values ≥0.90 indicated very strong correlation [44]. A multivariable regression analysis based on the backward stepwise variable selection technique was performed to create an inter-scale relationship model. The scales’ internal consistency (reliability) was evaluated using Cronbach’s alpha coefficients [45]. The statistical significance was set at *p* < 0.05.

### 3.7. Validity and Reliability/Rigor

The validity and reliability of the questionnaires (relating to resilience, individual performance, and hope) used in this study were established in previous publications. All scales were originally developed in English. However, the scales have been translated into Turkish for validity and reliability [40,41,43]. According to a review of the literature, resilience and individual performance consisted of one dimension, while two sub-dimensions explained hope. Cronbach’s alpha coefficients were calculated to examine the reliability of the Turkish version of all scales. In this study, Cronbach’s alpha values for resilience, hope, and individual performance were 0.87, 0.86, and 0.88, respectively. For the two sub-factors of the hope scale, pathway thinking and agency thinking, Cronbach’s alpha coefficients were 0.83 and 0.82, respectively.

## 4. Results/Findings

Descriptive statistics for demographic characteristics, resilience, hope, and individual performance are shown in Table 1. The average age of the 412 participants was 37.95 ± 4.08 (with median (range): 40.5 (20, 56)). Of these participants, 117 (28.4%) were between 40 and 49 years old. A total of 229 (55.6%) participants were women. Of the participants, 251 (60.9%) were married and 226 (54.9%) were physicians. Nurses constituted 16% of the sample; other health workers constituted 16.5%, and administrative staff constituted 12.4%. A total of 151 participants (36.7%) had 20 years or more experience, while 83 (20.1%) had 1–5 years of experience. A total of 146 (35.5%) employees were university graduates, and 188 (45.6%) had at least a master’s or doctoral degree.

The differences in the mean scale scores between the demographic characteristic groups were tested and the findings are shown in Table 2. Gender, age and professional seniority had a significant effect on the mean resilience. Age had a statistically significant effect on all scale means and a tendency to increase in scale means was detected as age increased. Men’s hope level was higher than women’s (*p* = 0.004). Gender had a significant effect on resilience, hope, and agency thinking. Men’s mean scores for resilience, hope, and agency thinking were higher than women’s. Profession only affected the agency thinking score, while professional seniority resulted in significant differences in all scale scores. The mean score increased as the amount of experience increased. All three education groups exhibited differences for all scales except resilience (*p* < 0.05). Age, marital status, professional seniority, and education were found to be important variables with regard to individual performance, which was considered as the dependent variable in this study.

Bivariate correlations between demographic variables, scale scores, and individual performance are shown in Table 3. Based on these findings, weak to moderate and significant correlations were observed between individual performance and age (r = 0.25, *p* < 0.001), resilience (r = 0.29, *p* < 0.001), and professional seniority (r = 0.26, *p* < 0.001), whereas moderate to strong and significant correlations were detected between individual performance and hope (r = 0.46, *p* = 0.001), agency thinking (r = 0.56, *p* = 0.001), and pathway thinking (r = 0.48, *p* = 0.001). On the other hand, moderate to strong and significant correlations were found between resilience and the level of hope (r = 0.73, *p* < 0.01) and its sub-factors (r = 0.54, *p* < 0.01 for agency thinking, r = 0.56 and *p* < 0.01 for pathway thinking, respectively). Gender, profession, and education were not significantly associated with employee performance (*p* ≥ 0.05). However, all of them were included in multivariate analyses.

### 4.1. Stepwise Regression Analysis

A stepwise regression analysis was performed to explain individual performance based on demographic variables, resilience, hope, pathway thinking, and agency thinking sub-factors. Demographic variables and the total scores of the two psychometric scales (resilience, hope, and its sub-factors) that were significantly correlated with individual performance in bivariate analyses were included in the multivariable regression analysis. Therefore, the age of the participants, as well as their gender, marital status, education level, profession, and professional seniority, were included in Model 1. Resilience, hope, pathway thinking, and agency thinking were added in Step 2 of the stepwise regression model.

According to the findings of the stepwise regression analysis, the first model was statistically significant (F = 12.65, *p* < 0.001). The demographic variables (age, gender, and profession) explained about 9% of the variance in individual performance (R^2^ = 0.09, F(3, 407) = 12.65, *p* < 0.001). The second model, which included age, gender, profession, resilience, hope, pathway thinking, and agency thinking, accounted for 35% of the variance in individual performance (R^2^ = 0.35, F(6, 404) = 43.44, *p* < 0.001). As a final model, standardized beta coefficients demonstrated that only age (β = 0.13, *p* = 0.003, 95% CI [0.26, 1.22]), gender (β = −0.10, *p* =0.016, 95% CI [−2.18, −0.23]), profession (β = 0.12, *p* =0.005, 95% CI [0.19, 1.07]), pathway thinking (β = 0.15, *p* =0.013, 95% CI [0.04, 0.33]), and agency thinking (β = 0.42, *p* < 0.001, 95% CI [0.36, 0.66]) were significant factors contributing to individual performance (Table 4).

### 4.2. Mediation Analysis

**Figure 2:**
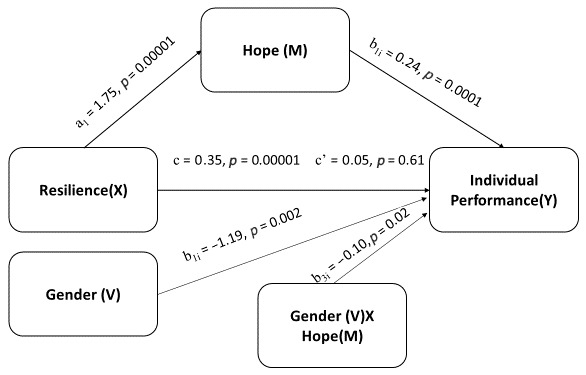
Path coefficients for simple mediated moderation analysis on individual performance.

The mediated moderation analysis was performed using the Hayes Process Macro with Model 14 (Figure 2).

Resilience significantly predicted the mediating variable, hope, E = 1.75, se = 0.083, t = 21.24, *p* = 0.00001, 95% CI (LLCI = 1.59, ULCI = 1.91). The explanatory power of the Hayes Process with model 14, which explained the predictive effect of resilience, hope, gender, and the interaction between gender and hope in terms of individual performance, was (R^2^) 0.25, F(4, 407) =33.12, *p* = 0.00001. Resilience did not significantly predict individual performance (outcome variable E = 0.05, se = 0.07, t = −0.74, *p* = 0.6179, 95% (LLCI = −0.19, ULCI = 0.09)). On the other hand, hope significantly predicted individual performance (E = 0.24, se = 0.03, t = 8.29, *p* = 0.00001, 95% CI (LLCI = 0.18, ULCI = 0.29)). Also, gender significantly predicted performance (E = −1.19, se = 0.52, t = −2.28, *p* = 0.002, 95 % CI (LLCI = −2.20, ULCI = −0.16)). In addition, gender and hope together significantly predicted performance (E = −0.097, se = 0.04, t = 2.42, *p* = 0.02, 95% CI (LLCI = 0.04, ULCI = 0.18)).

**Figure 3:**
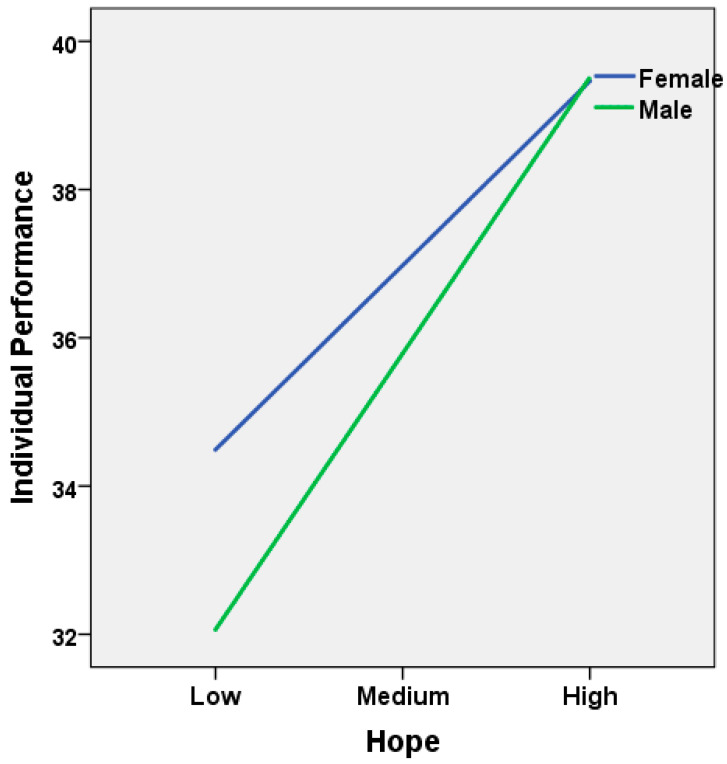
Moderating effect of gender and mediating effect of hope on individual performance.

In other words, the effect of hope on performance in women was significant (E = 0.20, se = 0.03, t = 5.76, *p* = 0.00001, 95% CI (LLCI = 0.13, ULCI = 0.26)). The effect of hope on performance was also significant in males (E = 0.29, se = 0.04, t = 8.03, *p* = 0.00001, 95% CI (LLCI = 0.22, ULCI = 0.36)). When hope was at low and medium levels, women outperformed men. However, as hope increased, men’s performance increased further and as a result, the difference between women and men disappeared when hope levels were high (see Figure 3).

The moderated mediation index was significant (E(index) = 0.17, BootSE = 0.08, BootLLCI = 0.02, BootULCI = 0.33). In other words, the moderating effect of gender in the mediation relationship was significant. In women, the indirect effect of hope, which was the mediating variable in the resilience–hope–performance relationship, was significant (M = 0.34, BootSE = 0.06, BootLLCI = 0.23, BootULCI = 0.46). In men, the indirect effect of hope, which was the mediating variable in the resilience–hope–performance relationship, was also significant (M = 0.51, BootSE = 0.07, BootLLCI = 0.37, BootULCI = 0.66). Comparing the indirect effect of female gender (Effect 1 value 0.34) with male gender (Effect 2 value 0.51), the indirect effect of hope on the resilience–hope–performance relationship was significantly different (E(contrast) = 0.17, BootSE = 0.08, BootLLCI = 0.02, BootULCI = 0.33).

## 5. Discussion

This study aimed to investigate the relationship between resilience and hope levels in healthcare workers who experienced the COVID-19 pandemic, the effects of resilience and gender on individual performance, the effects of hope levels on individual performance, and whether hope would mediate the effect of resilience on individual performance.

We accepted H1, which stated, “There is a relationship between the psychological resilience and hope levels of health workers”. The results of this research support the research findings of Gillespie et al. [21], Collins [10], Demetriou et al. [4], and Sadeghi vd. [33].

One of the most important results of this research is the acceptance of hypothesis H2, which states that “The level of resilience of health workers has significant effects on their individual performance”. In other words, a one-unit increase in the psychological resilience levels of healthcare professionals causes an increase of 0.35 times in the level of individual performance. This result is consistent with those of Demirsel, Erat & Kara [13]; Çam & Büyükbayram, [14] and Luthans et al. [7] 2005; and Luthans et al. [2]. Another important finding of this study is that the hope levels of health professionals have a significant effect on their individual performance. A one-unit increase in the hope levels of healthcare professionals created a 0.24-fold increase in individual performance. This shows that hypothesis H3 is supported, and this result is also consistent with the findings of Luthans et al. [7]; Luthans & Youssef, 2004 [1]; and Youssef & Luthans, 2007 [12]. Similarly, research has demonstrated that the hope levels of employees in different sectors exposed to the COVID-19 pandemic increase their performance [46]. In terms of increases in individual performance occurring as a result of psychological resilience and hope, this study is in line with findings of other studies by Luthans et al. [2]; Luthans et al. [6]; Sun et al. [8]; and Youssef and Luthans [12].

Hypothesis H4, which refers to “the indirect effect of the psychological resilience levels of healthcare professionals on their individual performance through hope” was not found to be significant. Therefore, when the hope variable was included as an explanatory factor in addition to resilience in the regression model, it was observed that resilience was not statistically significant. In our multivariate stepwise regression analysis, in the final model created for individual performance estimation, the previous moderator analysis supported our finding by showing that demographic variables of age, gender, and occupation, as well as two sub-factors of hope, affected individual performance. Consequently, the findings of the multivariate stepwise regression analysis suggested that only age (β = 0.13), gender (β = −0.10), profession (β = 0.12), pathway thinking (β = 0.15), and agency thinking (β = 0.42) were significant factors contributing to individual performance (R^2^ = 0.35). Luthans et al. [7] found that supportive leadership resulted in a positive correlation between the hope and resilience of employees and, thus, improved their performance (hope, r = 0.17, resilience, r = 0.24). This finding highlights the need for further research.

In their study of 1000 nurses, Sun et al. [8], found significant relationships between individual performance and age, years spent working, self-efficacy, hope, and resilience. In particular, considering the M1 model in the structural equation model they used, the significant effect of the PsyCap Questionnaire with four components (efficacy, hope, resilience and optimism) on performance (β = 0.52) provided the perfect model fit.

Additionally, Luthans et al. [6], in their clinical controlled trial study, noticed that the cross-section of managers’ reported levels of PsyCap was significantly related to their performance. Considering the results of the study by Youssef & Luthans [12], a moderate and significant (r = 0.52) correlation between psychological resilience and hope level was observed. Additionally, education, experience, industry sector, and hope level were important variables that affected performance. In this sense, it can be said that the common variables that have a significant impact on individual performance in our stepwise regression results and the findings of Youssef & Luthans [12] are the level of professional experience and the level of hope.

A study that included 214 healthcare workers found that in model 1, gender, age, occupation (which encompassed doctors, nurses and other healthcare professionals), and having a child were significant predictors of psychological resilience [47]. In model 2, age, occupation, worry about becoming infected by the virus, and quality of sleep significantly affected the psychological resilience of healthcare professionals. Yıldırım and Güler [48] conducted a study involving 168 healthcare workers and found that coronavirus anxiety had a significant positive correlation with fear of COVID-19 (r = 0.70) and significant negative correlations with hope (r = −0.16) and resilience (r = −0.41). Additionally, a significant positive correlation was detected between hope and resilience (r = 0.44).

Our current study revealed that the hope level of males was higher than that of females. Gender also had a significant impact on resilience, hope, and agency thinking. On average, males’ resilience, hope, and agency thinking scores were higher than those of females. The explanatory power of Hayes Process model 14, which described the predictive effect of resilience, hope, gender, and the interaction between gender and hope on individual performance (R^2^) was 0.25. The significant moderating effect of gender in the mediation relationship also added an original aspect to the study. The current research findings also support hypothesis H5, which states that “When women have low or moderate hope, they are expected to outperform men”. Gender played a moderating role between hope and performance, indicating that gender difference was evident only when hope levels were low but disappeared in high hope conditions. The significant moderating effect of gender in the mediation relationship added an original feature to the study as well. Women outperformed men when hope was at low to moderate levels. However, as hope increased, men’s performance increased more and as a result, the difference between men and women disappeared when hopes were high. These findings are consistent with those of [20,32,49]; Ahlawat and Budhiraja [32]; Lian et al. [49]; and Snyder et al. [20]. On the other hand, these results are similar to those of Gisinger et al. [50]; Baguri et al. [51]; Sadeghi et al. [33]; and Tarhan [41]. The results of this study may be a step in the right direction for future research wishing to investigate the effects of gender on psychological factors and performance. Considering the importance of cultural differences, this study is important for Türkiye, which comprises both collectivist [52] and individualistic cultures. In Turkish culture, where patriarchal ideology is dominant, as in various countries around the world, educated and working women are not able escape traditional gender roles. This situation, which can create a balance and inequality problem [53] in many areas, especially when it comes to education, work, and family life, is an advantage when it comes to hope. As this research indicates, it is not surprising that women’s individual performance is better than men’s in situations where hope is low or moderate, because a woman always has the ability and self-discipline to think of and do many things at the same time. This may include completing traditional tasks such as housework and childcare, on the one hand, and fulfilling work and professional responsibilities, on the other hand, in both positive or negative situations. This also applies to healthcare workers who have much more responsibilities at work. The presence of hope can perhaps be thought of as a feature of being a woman.

It should be kept in mind that the hopes of men and women are different from each other and the gender roles in the culture one lives in are contributing factors. For example, considering the effects of traditional gender roles in the United States, where men are expected to produce higher levels of hope, research has shown that there is no gender difference in the hope level [20]. In the literature, the findings of the focus group study by Tarhan [41], in which gender roles affect hope, support our study findings. Our findings were also consistent with Baguri et al.’s [51] findings. They found that gender had a moderate effect on teacher resilience during the pandemic. In the study, male teachers had a higher level of teacher resilience with a higher tendency toward hope than female teachers, which is one of the important supporting results of this study. In line with this finding, Sadeghi et al. [33], in a study of Iranian adolescents, found that male adolescents exhibited higher levels of self-differentiation, resilience, and hope than female adolescents, which is reflective of the parenting style in Middle Eastern culture. While the tendency to own one’s thoughts and feelings without the need to conform to others’ expectations contributes to greater resilience in boys, the tendency to be emotionally close to others contributes to increased resilience in girls [33].

It can be said that hope has more importance than resilience when it comes to increasing individual performance. Our findings show that employees with high levels of hope and resilience are more likely to recover from stressful situations and achieve better individual performance. The results of this study make a valuable contribution to the psychology, organizational behavior, and health management literature.

It should be noted that compassion fatigue can arise when resilience and hope are excessively emphasized, leading individuals to overextend their limits and jeopardize their mental and physical health. This issue is particularly prevalent in the healthcare and caregiving sectors. Therefore, strategies that support individuals in establishing healthy boundaries for both others and themselves are essential. When it is disconnected from reality, hope can lead to toxic optimism, causing individuals to remain in harmful situations. Thus, hope should be approached through the lens of “realistic hope”, which involves a pragmatic evaluation of circumstances and, when necessary, the development of alternative paths or strategies for withdrawal. In conclusion, resilience and hope must be addressed in a context-sensitive manner to empower individuals to protect themselves and navigate challenges more effectively.

The special situation of Istanbul, resulting from its dense population and its important role in the Turkish economy, as well as the large number of people who had to work, were the main factors that negatively affected the impact of the measures taken by the government during the pandemic period. The government’s ability to protect public health and healthily manage the pandemic was affected by the number of patient beds in city hospitals, which had been built over the years to reach sufficient capacity. The provision of adequate vaccines and the achievement of the target number of vaccinations allowed the pandemic to be managed in a controlled manner.

This study has important implications for advancing the field of psychosocial assessment of healthcare workers in times of health crisis. The effects of hope and resilience on employee performance as psychological capital that can help all healthcare managers and employees, especially in times of crisis such as the COVID-19 outbreak, can make many scientific contributions to developing health policy and management strategies. In addition, the fact that the psychological resilience, hope, and well-being of health workers will not only improve their health but also the effectiveness of the overall health system can be seen as another social contribution of the study. Organizations should focus more on hope and resilience by fostering positive attitudes in both employees and managers, allowing them to manage crises better. The adoption of psychological approaches in institutions will enable the development of crisis management teams and psychosocial intervention programs.

### Limitations

This study had some limitations. The data were collected from authorized healthcare professionals (who agreed to participate voluntarily) in Istanbul, Türkiye’s most populous province; as such, the sample did not include national representation. Secondly, because the study design was cross-sectional, it was not possible to draw definite conclusions about the cause–effect relationships between the investigated variables. Longitudinal studies are needed to determine the direction of these relationships. Thirdly, this study used a convenience sampling approach for data collection, which was another limitation. Because the data were collected after healthcare workers in Türkiye had received two doses of the COVID-19 vaccination, we could not monitor temporal changes in responses.

## 6. Conclusions

This study offers a practical and theoretical perspective on the effects of hope and resilience on employee performance. Hope and resilience are forms of psychological capital that can help all managers and employees working in the healthcare field, especially during crisis periods such as the COVID-19 pandemic. We also revealed the direct and indirect effects of psychological resilience and hope levels on the individual performance of healthcare professionals during the pandemic period using a robust statistical analysis approach. Examining the roles of personal resources, such as hope and resilience between stressors caused by COVID-19 and individual performance, is important for providing benefits for employees which will help them to protect their psychological health against the harmful effects of such crises.

The results of this study are consistent with the predictions of the hope theory and job demand source (JDR) models. According to Snyder’s hope theory [11], individuals can motivate themselves to plan the pathways leading to their goals. The pandemic has been challenging for healthcare workers, who face difficulties associated with excessive workloads and insufficient resources. The job demand source (JDR) model states that employees need resources to cope with their demanding work routines. In the JDR model, employees who use their resources as a buffer against daily workplace difficulties that negatively affect their performance are likely to achieve their goals, as they have more substantial psychological resources [54]. Therefore, hard work is associated with greater access to resources and ultimately improves performance.

Health institutions are labor-intensive institutions that are important to society. Employees are very important to the success of these organizations, which were established to meet the health service needs of society and fulfill health-related goals. The events occurring in an employee’s social environment continually shape his or her hope and resilience. Thus, managers should implement these measures to reduce stress factors in employees.

## Figures and Tables

**Table 1:** Descriptive statistics of the study parameters (N = 412).

Demographic Characteristics	N (%)	Professional Seniority (Years)	N (%)
Gender		1–5	83 (20.1)
Female	229 (55.6)	6–10	68 (16.5)
Male	183 (44.4)	11–15	58 (14.1)
Age		16–20	52 (12.6)
20–29	91 (22.1)	20 and over	151 (36.7)
30–39	108 (26.2)	Education	
40–49	117 (28.4)	Associate of Science	78 (18.9)
50 and over	96 (23.3)	Bachelor’s Degree	146 (35.5)
Marital Status		Master’s Degree	188 (45.6)
Married	251 (60.9)		
Single	161 (39.1)		
Profession			
Physician	226 (54.9)		
Nurse	66 (16.0)		
Other health personnel	68 (16.5)		
Administrative staff	51 (12.4)		
Missing	1 (0.2)		
**Scales**	x **(sd)**	**Range**	
Resilience	20.27 (5.26)	6–30	
Individual performance	36.54 (5.87)	12–45	
Hope	68.79 (12.70)	25–96	
Pathway thinking	25.34 (4.83)	7–32	
Agency thinking	24.4 (4.86)	4–32	

**Table 2:** Comparisons of mean scale scores based on the demographic characteristics.

Demographic Characteristics	Scale				
	Resilience	Individual Performance	Hope	Pathway Thinking	Agency Thinking
	x±sd/median (IQR)				
Gender					
Female	19 (6.5)	37 (8)	67.17 ± 12.4	25 (7)	25 (6)
Male	21 (8)	37 (8)	70.81 ± 12.8	27 (8)	26 (6)
*p*-value	<0.001	0.707	0.004	0.062	0.011
Age					
20–29	18.9 ± 4.9 *	33.9 ± 6.1 *	64.85 ± 11.9	23.93 ± 4.6 *	22.61 ± 4.7 *
30–39	19.04 ± 5.2 ^#^	36.12 ± 6.1	65.44 ± 12.8 *	24.28 ± 5.1	23.19 ± 4.9
40–49	20.29 ± 5.2	37.63 ± 5.7 *	69.35 ± 13.1	25.90 ± 4.6 *	24.74 ± 4.8
50 and over	22.81 ± 4.7 *^#^	38.14 ± 4.4	75.60 ± 9.6 *	27.16 ± 4.1	26.98 ± 3.6 *
*p*-value	<0.001	<0.001	<0.001	<0.001	<0.001
Marital Status					
Married	20 (7)	37 (8)	69.39 ± 12.6	26 (6)	26 (6)
Single	19 (7)	36 (10)	67.85 ± 12.8	25 (7)	25 (6)
*p*-value	0.654	0.048	0.233	0.135	0.011
Profession					
Physician	20 (7)	36.30 ± 6.2	70.04 ± 12.4	25 (0)	26 (6) *^#^
Nurse	20 (6)	36.92 ± 5.0	68.90 ± 12.5	26 (5)	25 (6)
Other health personal	19 (6.75)	36.89 ± 5.8	67.20 ± 12.1	25 (6)	24 (6) ^#^
Administrative staff	20 (8)	36.52 ± 5.4	64.41 ± 14.6	26 (8)	24 (8) *
*p*-value	0.297	0.831	0.077	0.676	0.017
Professional Seniority (years)					
1–5	19.20 ± 5.1 *^#^	34.26 ± 6.4 *^#^	65.33 ± 11.0 *	24.07 ± 4.2 *	22.98 ± 4.6 *
6–10	18.58 ± 5.4	34.64 ± 6.5	64 ± 14.2 ^#^	23.57 ± 5.6 ^#^	22.08 ± 5.5 ^#^
11–15	20.06 ± 4.6	36.63 ± 4.7	67.5 ± 11.0	25.06 ± 3.9	23.81 ± 3.7
16–20	19.69 ± 6.3 ^#^	38.42 ± 5.6 *	69.8 ± 15.7	26.11 ± 5.3	24.84 ± 5.4
20 and over	21.88 ± 4.6 *	37.94 ± 5.0 ^#^	73 ± 11.0 *^#^	26.66 ± 4.3 *^#^	26.26 ± 4.0 *^#^
*p*-value	<0.001	<0.001	<0.001	<0.001	<0.001
Education					
Associate of Science	19.44 ± 5.1	35 (9) *^#^	65.65 ± 13.3 *	24 (8) *	23 (5) *^#^
Bachelor’s Degree	20.30 ± 5.5	37 (7) ^#^	68.93 ± 13.2	26 (7)	25 (6) ^#^
Master’s Degree	20.57 ± 5.0	37 (8) *	69.98 ± 11.8 *	26 (6) *	26 (6) *
*p*-value	0.280	0.031	0.041	0.019	<0.001

* and #: The difference between subcategories with the same sign is statistically significant.

**Table 3:** Bivariate correlations between demographic characteristics, resilience, hope, and individual performance.

Variable	1	2	3	4	5	6	7	8	9	10	11
1. Age	-										
2. Gender	0.29 **	-									
3. Marital status	−0.45 **	−0.11 *	-								
4. Education	0.44 **	0.09	−0.16 **	-							
5. Profession	−0.23 **	−0.19 **	0.07	−0.48 **	-						
6. Professional Seniority	0.89 **	0.17 **	−0.45 **	0.37 **	−0.16 **	-					
7. Resilience	0.27 **	0.20 **	−0.02	0.06	−0.08	0.23 **	-				
8. Hope	0.32 **	0.16 **	−0.06	0.11 *	−0.11 *	0.28 **	0.73 **	-			
9. Individual performance	0.25 **	−0.01	−0.09 *	0.09	0.01	0.26 **	0.29 **	0.46 **	-		
10. Agency thinking	0.35 **	0.13 *	−0.13 *	0.18 **	−0.16 **	0.33 **	0.54 **	0.81 **	0.56 **	-	
11. Pathway thinking	0.26 **	0.09	−0.07	0.13 **	−0.05	0.25 **	0.56 **	0.83 **	0.48 **	0.74 **	-

* *p* < 0.05, ** *p* < 0.01.

**Table 4:** Stepwise (backward) regression analysis of demographic variables and total scale scores with regard to individual performance.

	Model 1			Model 2		
Predictors	B	β	95% CI	B	β	95% CI
Age	1.66 **	0.31 **	(1.13, 2.20)	0.74 *	0.13 *	(0.26, 1.22)
Gender	−1.32 *	−0.11 *	(−2.47, −0.16)	−1.20 *	−0.10 *	(−2.18, −0.23)
Profession	0.39	0.07	(−0.12, 0.90)	0.63 *	0.12 *	(0.19, 1.07)
Resilience				−0.02	−0.02	(−0.14, 0.08)
Hope				−0.05	−0.12	(−0.14, 0.03)
Pathway thinking				0.18 *	0.15 *	(0.04, 0.33)
Agency thinking				0.51 **	0.42 **	(0.36, 0.66)
R^2^	0.09			0.35		
R^2^ change	0.10			0.34		
F statistics	12.65 **			43.44 **		

* *p* < 0.05, ** *p* < 0.01.

## Data Availability

The data in this study can be provided upon request by sending an e-mail to the corresponding author.
